# A Study on the Effect of the Substituent against PAK4 Inhibition Using In Silico Methods

**DOI:** 10.3390/ijms23063337

**Published:** 2022-03-19

**Authors:** Hye Ree Yoon, Chong Chul Chai, Cheol Hee Kim, Nam Sook Kang

**Affiliations:** 1 Graduate School of New Drug Discovery and Development, Chungnam National University, 99 Daehak-ro, Yuseong-gu, Daejeon 34134, Korea; hyeree7775@naver.com; 2 Pharos iBio Co., Ltd. #1408, 38 Heungan-daero 427, Dongan-gu, Anyang-si 14059, Korea; ccchai1@gmail.com (C.C.C.); cheolheekim@pharosibio.com (C.H.K.)

**Keywords:** PAK4, molecular mechanics, fragment molecular orbital, molecular electrostatic potential

## Abstract

The intrinsic inductive properties of atoms or functional groups depend on the chemical properties of either electron-withdrawing groups (EWGs) or electron-donating groups (EDGs). This study aimed to evaluate in silico methods to determine whether changes in chemical properties of the compound by single atomic substitution affect the biological activity of target proteins and whether the results depend on the properties of the functional groups. We found an imidazo[4,5-b]pyridine-based PAK4 inhibitor, compound **1**, as an initial hit compound with the well-defined binding mode for PAK4. In this study, we used both experimental and in silico methods to investigate the effect of atomic substitution on biological activity to optimize the initial hit compound. In biological assays, in the case of EWG, as the size of the halogen atom became smaller and the electronegativity increased, the biological activity IC_50_ value ranged from 5150 nM to inactive; in the case of EDG, biological activity was inactive. Furthermore, we analyzed the interactions of PAK4 with compounds, focusing on the hinge region residues, L398 and E399, and gatekeeper residues, M395 and K350, of the PAK4 protein using molecular docking studies and fragment molecular orbital (FMO) methods to determine the differences between the effect of EWG and EDG on the activity of target proteins. These results of the docking score and binding energy did not explain the differences in biological activity. However, the pair-interaction energy obtained from the results of the FMO method indicated that there was a difference in the interaction energy between the EWG and EDG in the hinge region residues, L398 and E399, as well as in M395 and K350. The two groups with different properties exhibited opposite electrostatic energy and charge transfer energy between L398 and E399. Additionally, we investigated the electron distribution of the parts interacting with the hinge region by visualizing the molecular electrostatic potential (MEP) surface of the compounds. In conclusion, we described the properties of functional groups that affect biological activity using an in silico method, FMO.

## 1. Introduction

Chemical properties depend on the intrinsic inductive properties of the atoms or functional groups of the compound. This property refers to the ability of atoms or functional groups in a compound to withdraw or donate electrons [1]. Electron-withdrawing groups (EWGs) typically include halogen atoms, and many drugs and drug candidates under clinical development are found to contain these. Incorporation of halogen atoms in a drug is known to alter physicochemical properties, thus improving its oral absorption, the blood-brain barrier permeability, and increasing cell membrane permeability [2,3,4]. Therefore, the addition of a halogen atom is used in most cases for the hit to lead the development and lead optimization; however, its use is commonly restricted to produce steric properties rather than stabilizing effects [5]. Conversely, electron-donating groups (EDGs) include functional groups such as CH_3_ and OCH_3_ [6]. In small molecules, the methyl group at an appropriate site frequently plays an important role in increasing the hydrophobicity. It can also significantly alter the bioavailability and efficacy of bioactive molecules as well as their interaction with the receptors [7,8].

In this study, we investigated whether changes in the chemical properties caused by a single atom affect the biological activity of the target protein. Ultimately, the aim of this study was to evaluate the properties of the functional groups needed to affect biological activity using in silico methods.

Here, p21-activated kinase 4 (PAK4) protein was selected as the target protein. PAK4 overexpression or activation in human tissues is often associated with cancer and oncogenic transformation [9,10,11,12]. PAK4 overexpression correlates with various cancers, including breast cancer [13], ovarian cancer [14], pancreatic cancer [15], prostate cancer [16], gastric cancer [17], and gliomas [18]. In a previous study, we discovered an inhibitor with a unique scaffold that inhibited PAK4 activity despite its small size. In this paper, as compound **1** [19], this inhibitor is an initial hit compound for PAK4. The binding mode of the PAK4 inhibitor was revealed using X-ray crystallography [19], where the scaffold 3H-imidazo[4,5-b]pyridine forms hydrogen-bonding interactions between the backbone nitrogen and carbonyl oxygen of L398 at 2.8 Å and 2.6 Å distance, respectively. Moreover, it forms the same binding interactions with the atoms of the side chain of L447 at 3.5 Å and 3.7 Å distance. The bromine group in the pyridine of the scaffold forms van der Waals interactions with the side chain of A348 (4.1 Å), side-chain carbon of M395 (4.4 Å), and side-chain gamma carbon of K350 (4.7 Å). M395 and K350 interacting with the bromine group are the gatekeeper residues of PAK4, and the K350 mutation is known as the kinase-dead mutant PAK4 (K350M) [20,21]. Therefore, we hypothesized that the presence of a bromine moiety would affect the biological activity of PAK4. Based on the clear bonding mode, we assessed compounds with EWGs such as Cl and F instead of bromine groups and with EDGs such as CH_3_, having an opposite inductive effect.

To analyze the influence of EWGs or EDG substitution on the biological activity, molecular docking and fragment molecular orbital (FMO) methods were used. Molecular docking in structure-based drug design has been widely used and is the most common method for determining pose ranking using the force field-based scoring function [22,23]. All molecular docking methods require a scoring function to rank various candidates, and these scoring functions are classified into empirical, knowledge-based, or molecular mechanics [24]. The molecular docking predicts the binding mode and affinity of small molecules within the binding site of the target protein. It is also a useful method for understanding key physicochemical characteristics.

Among the in silico methods, the FMO method is currently widely used to determine target protein-ligand binding properties and to design new inhibitors for drug discovery. This method is used to assign suitable ligand binding poses among several similarly scored poses in virtual screening using molecular docking; it is also employed to assign the optimal fragment-binding pose present in X-ray crystal structures for fragment-based drug discovery [25]. The FMO method is less time-consuming than conventional quantum mechanics (QM) methods as it involves dividing a macromolecule into smaller fragments and then calculating the QM of each fragment [26,27,28,29,30]. This method classifies and quantifies the energy values of each interacting fragment in the protein-ligand complex state. The interaction between the two fragments is characterized by electrostatic, exchange repulsion, charge transfer, and dispersion interactions [31]. After the FMO calculation, the pair-interaction energy (PIE), representing the sum of interaction energies between two fragments calculated in FMO, was analyzed [25,30,32,33]. The key difference between FMO and molecular docking from molecular mechanics methods is that the electrons account for polarization and charge transfer effects [26,34]. In addition, we visualized the molecular electrostatic potential (MEP) surface to observe electron distribution [35]. The MEP surface is a crucial tool for understanding other molecular interactions within the small molecules. In the current study, it was confirmed through an in silico study that the difference in compound properties due to a single atomic substitution on the inhibitor affects the biological activity of the target protein.

## 2. Results

**Figure 1:**
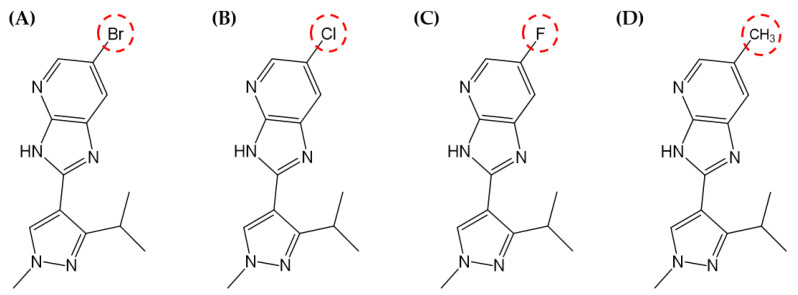
2D structures of compounds. (**A**) compound **1**, (**B**) compound **2**, (**C**) compound **3**, and (**D**) compound **4**. The red circles represent the substituted groups.

The compounds used in the analysis were based on compound **1**, a ligand of Protein Data Bank Code 5I0B [19]. Compounds **2** (Otava ID 26753422), **3** (Otava ID 26753421), and **4** (Otava ID 26753420) were purchased from Otava Ltd. (Vaughan, Ontario, L4K 0C3, Canada, https://otavachemicals.com/, accessed on 23 February 2022), and the compound structure is as shown in Figure 1.

Compounds **1**–**3** have various substituted EWGs with varying atomic sizes, electronegativities, and electron affinities. In contrast, the substituted group on compound **4** was an EDG. We performed an in vitro assay to confirm that atomic substitution affected the biological activity of the target protein. The IC_50_ (nM) values against PAK4 were 5150 nM for compound **1**, 8533 nM for compound **2**, and more than 30,000 nM for compounds **3** and **4** (see Table 1).

### 2.1. Molecular Docking Studies against PAK4

**Figure 2:**
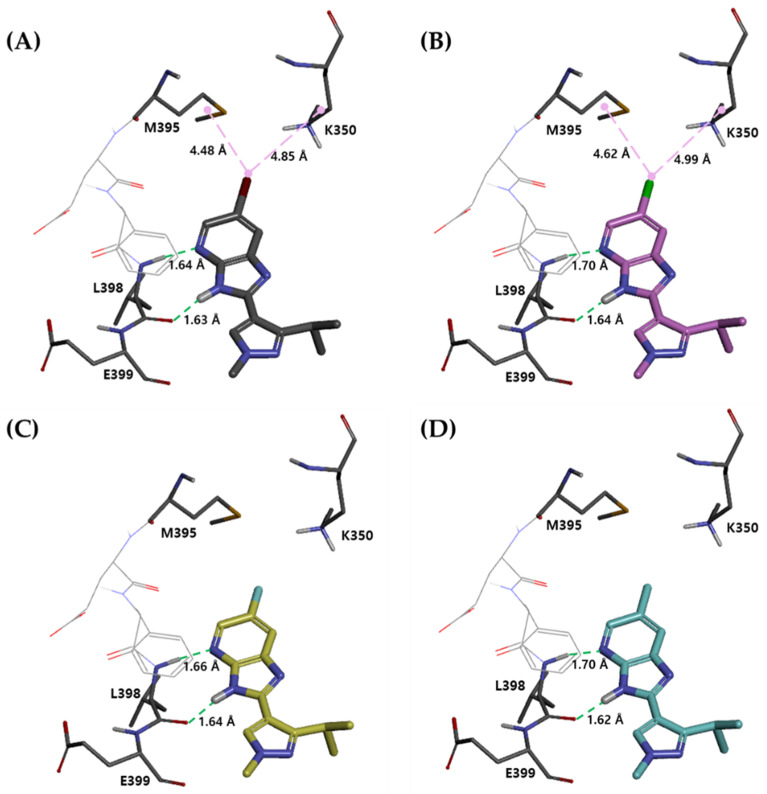
Binding modes of (**A**) compound **1**, (**B**) compound **2**, (**C**) compound **3**, and (**D**) compound **4** inside the PAK4 binding pocket. Ligands are shown as stick models, while hydrogen bonding interactions are displayed as green dashed lines, and van der Waals interactions are displayed as a pink line. Hydrogen bond distances are provided in Å.

The binding mode of the compounds was predicted using the reference ligand of PAK4, Protein Data Bank code 5I0B [19]. In the binding mode of each compound obtained through the molecular docking study, there was no significant difference between compounds **1**–**3** with EWG and compound **4** with EDG. As shown in Figure 2, a common feature of the binding mode is that the compounds form hydrogen-bonding interactions between the L398 backbone of the PAK4 hinge region and 3H-imidazo[4,5-b]pyridine. In the hydrogen-bonding interaction, the distance between the hydrogen donor of the L398 backbone and the compounds was 1.64–1.70 Å, and the distance between the hydrogen acceptor of the L398 backbone and the compounds was 1.62–1.64 Å. Among the compounds **1**–**3** with EWG, compounds **1** and **2** with Br and Cl, respectively, form van der Waals interactions with M395 side chains at 4.48 Å and 4.62 Å, and K350 side chains at 4.85 Å, 4.99 Å distances, respectively. In conclusion, the binding mode results obtained through molecular docking were insufficient to explain the difference in effects between EWG and EDG.

We also analyzed molecular docking scores and binding interaction energies (kcal/mol) using the calculated binding energy scores from the Discovery Studio module in the binding mode (see Table 1). The docking scores for the binding modes were 59.27, 56.95, 54.00, and 58.14 for compounds **1**, **2**, **3**, and **4**, respectively. The two limitations were as follows: First, the docking score results were insufficient to explain the biological activity. As shown in Table 1, compound **1**, with an IC_50_ of 5150 nM, had the highest docking score of 59.27, while the compound with the second-highest docking score of 58.14 was compound **4**, which was an inactive compound. Second, the binding energy results did not show any correlation with the docking score. The binding energies for compounds 1 to 4 were −23.78, −27.14, −25.16, and −22.78 kcal/mol, respectively. When comparing the docking score results of the four compounds, compound **1**, with the biological activity of 5150 nM, had a high score of 59.27. However, the binding energy of compound **1** was –23.78 kcal/mol, and compound **2** with 8533 nM was −27.14 kcal/mol, which was predicted to have more stable binding interactions than other compounds.

In conclusion, the docking study results were insufficient to explain the biological activities of the compound **1** series. The reason for the above results is that the scoring function implemented in the docking program is made through various assumptions and simplifications; therefore, it does not fully explain the various physical phenomena that determine molecular recognition for the complex [36]. Indeed, docking scores from molecular docking often provide results that do not correlate with the experimental binding affinity [37,38,39].

### 2.2. Pair-Interaction Energy Analysis

**Figure 3:**
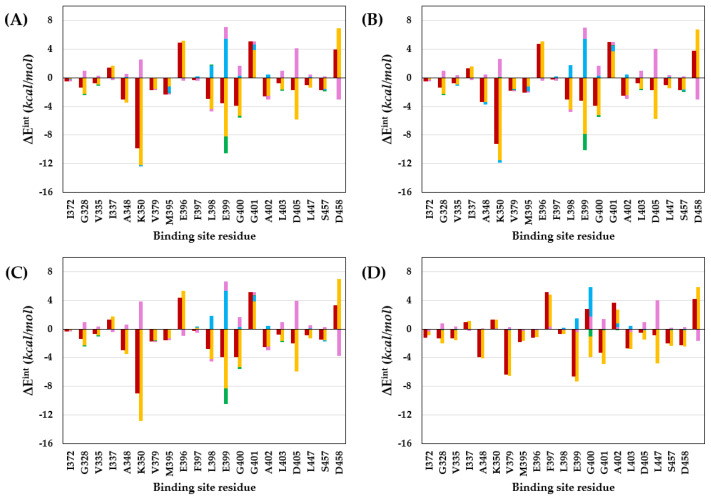
PIE calculated at the FMO. (**A**) compound **1**, (**B**) compound **2**, (**C**) compound **3**, and (**D**) compound **4**. In the graph, pair-interaction energy (E^int^), electrostatic (E^es^), exchange-repulsion (E^ex^), charge transfer (E^ct^), dispersion (E^di^), and solvation energy are shown in red, yellow, sky blue, green, dark blue, and pink colors, respectively.

**Figure 4:**
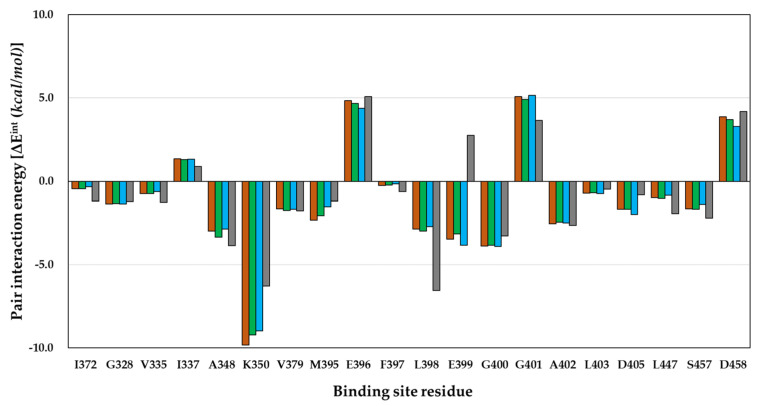
Comparison of calculated PIE in the FMO of compounds **1**–**4**. Compounds **1**, **2**, **3**, and **4** are shown in brown, green, light blue, and gray, respectively.

We performed PIE analysis after FMO calculations to detect important interactions between protein residues and compounds within the binding site. The PIE is the summation of the electrostatic, exchange repulsion, charge transfer, dispersion, and solvation energies. PIE indicates the strength of the interaction between the ligand and target protein residues within the complex and not the difference in the energies of the free and bound ligands. According to Table 1 and Figure 3, the energies between each compound in the residue within 5.0 Å distance, which is the van der Waals interaction region, were analyzed in the PIE analysis. In the analysis of the results, the interactions above the absolute PIE value of 3.0 kcal/mol were considered crucial, as reported [29]. As a result of PIE analysis, the energy value between the PAK4 protein residues and compounds is shown in Table 2 and Figure 4, where the energy difference between each compound and M395 and K350 is indicated. As the difference in the biological activity of the target protein depends on the size of the halogen atom and its electronegativity [40], a difference in the interaction between M395 and K350 was anticipated for compounds **1** to **3**. In addition, a difference in the distance between the halogen atom of each compound and K350 was observed in the binding mode obtained from molecular docking studies. In this study, the substituted atoms included an EDG and EWG, where we attempted to explain the difference in biological activity due to the influence of EWG and EDG. Therefore, it was suggested that the effects of EDG and CH_3_ were insufficient to explain the size or electronegativity, similar to that of EWG. We hypothesized that there might be residues within the binding site that support the biological activity of EWG and EDG. This is because, in the binding mode structure of the X-ray crystal ligand (compound **1**), the Br atom does not form a halogen bond but a van der Waals interaction, and in the case of CH_3_, hydrophobic interaction is possible. Residues showing a difference of more than 3 kcal/mol between the EWG and EDG in the PIE results were L398 and E399 (hinge region residues), but not K350 (gatekeeper residue).

**Figure 5:**
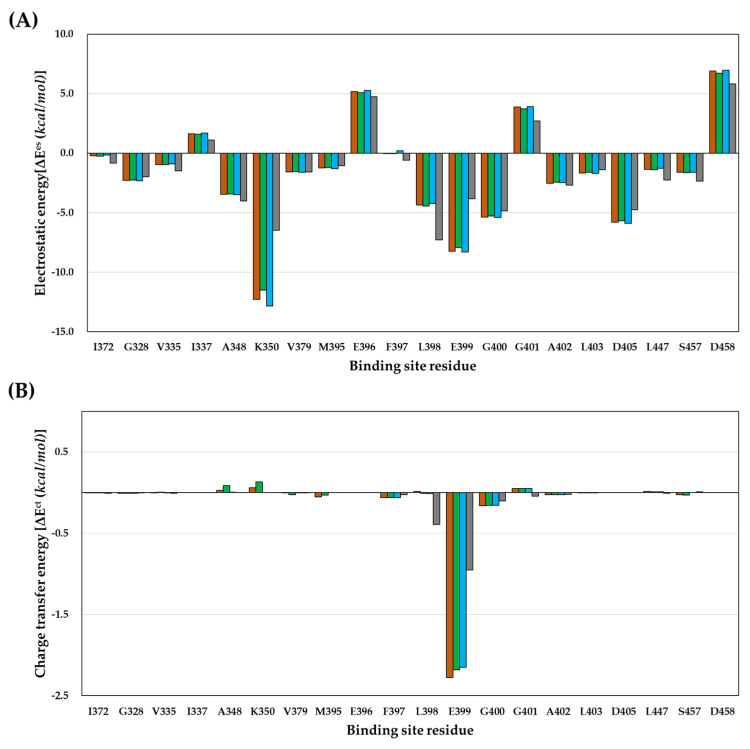
Comparison of calculated energies in the FMO of compound **1**–**4**. (**A**) Electrostatic energies (E^es^), (**B**) charge transfer energies (E^ct^). Compounds **1**, **2**, **3**, and **4** are shown in brown, green, light blue, and gray, respectively.

Interestingly, the energy patterns in Figure 4 were opposite to those of L398 and E399 in compounds **1**–**3** with EWG and in compound 4 with EDG. Here, L398 is a residue for the hydrogen-bonding interaction at the hinge region of PAK4, and E399, which is n+1 and also depends on the properties of the compound. The main energy values showing a different pattern between the EWG and EDG were the electrostatic and charge transfer energies. These energies were not determined in a molecular docking study [26]. The electrostatic and charge transfer energies are associated with polar interactions and hydrogen bonds [41]. The results of electrostatic energy and charge transfer energy between each compound and residue are shown in Table 3 and Table 4 and Figure 5.

Compound **4**, having an EDG, interacted strongly with the L398 residue compared to the other compounds. The electrostatic energies of compounds **1** to **4** for interaction with L398 were −4.37, −4.44, −4.24, and −7.28 kcal/mol, and the charge transfer energies were 0.02, −0.01, −0.01, and −0.39 kcal/mol, respectively. The difference in electrostatic energy and charge transfer energy of compound **4** were −2.91 and −0.41 kcal/mol, respectively, compared to those of compound **1**. Additionally, the electrostatic energies of compounds **1** to **4** for interaction with E399 residue were −8.26, −7.92, −8.30, and −3.85 kcal/mol, and the charge transfer energies were −2.28, −2.18, −2.15, and −0.95 kcal/mol, respectively. The difference in electrostatic energy and charge transfer energy of compound **4** were 4.41 and 1.33 kcal/mol compared to those of compound **1**. Briefly, compound **4** with EDG had stronger electrostatic energy and charge transfer energy than compounds **1**–**3** with EWG during interaction with L398; therefore, the difference in the PIE value, which is the total energy, showed stronger energy by −3 kcal/mol or more. However, compound **4** with EDG had weaker electrostatic energy and charge transfer energy than compounds **1**–**3** with EWG during interaction with E399; therefore, the difference in PIE value, the summation value, showed weaker energy by more than 5 kcal/mol. In conclusion, the explanation of the biological activity of compound **4** for PAK4 with EDG interactions could describe why compound **4** had strong hydrogen-bonding interactions with L398 in the hinge region but was inactive due to repulsion between compound **4** and E399. We also calculated the MEP and observed a difference in the electron density of the portion interacting with the hinge region due to substitutions with EWG or EDG.

### 2.3. Molecular Electrostatic Potential Surface

**Figure 6:**
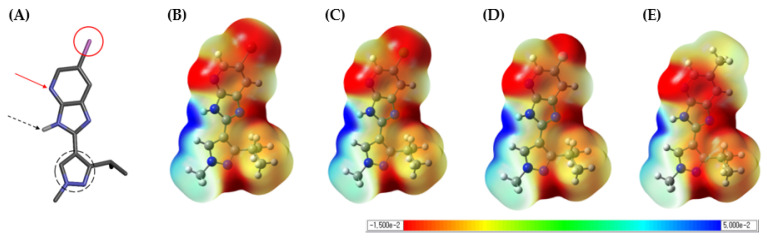
Compound 3D structure and the 3D plots of MEP surface. (**A**) Pink atom is indicated by a red circle as an atomic substitution part. The N atom of the pyridine on the scaffold is indicated by a red arrow. The NH atom of imidazole in the scaffold is indicated by a black dotted arrow, and the pyrazole is indicated by a black dotted circle. (**B**) Compound **1**, (**C**) compound **2**, (**D**) compound **3**, and (**E**) compound **4** computed at the DFT/B3LYP/6-311G++ (3df) level of theory (0.004 a.u.).

The MEP surface is related to the electronic density and is used as a highly beneficial descriptor for the determination of sites for electrophilic attack, nucleophilic reactions, and hydrogen-bonding interactions [42]. The MEP surface was calculated using quantum chemical calculations. Density functional theory (DFT) has become the method of choice for studying large systems due to the balance of accuracy and efficiency [43]. Among the DFT, B3LYP/6-311++G(3df) level of theory will be reliable and a good choice to study such cases compared to the most computationally expensive methods, such as G4MP2. The MEP surface was calculated using the B3LYP/6-311G++ (3df) optimized geometry [44]. Depending on the binding mode, the scaffold of the compound exhibits hydrogen-bonding interactions with the L398 backbone of the hinge region. In the imidazo[4,5-b]pyridine scaffold, pyridine shows a hydrogen-bonding interaction with the backbone hydrogen donor of L398, and imidazole shows a hydrogen-bonding interaction with the backbone hydrogen acceptor of L398. The MEP surface [45] results are shown in Figure 6, which shows the difference in electron density between the EWG and EDG depending on the atom substitution. In the case of compounds with EWGs, a difference in electron density was seen depending on the size and electronegativity of Br, Cl, and F, which explains the difference in the interaction energy between each compound and M395 and K350 in the PIE results. Second, the difference between the compounds with EWG and EDG was shown at the hinge region and hydrogen-bonding interactions with the scaffold. The scaffold was fused with pyridine and imidazole, and the electron density at the N atom of pyridine was higher in EDG than in EWG. This result explained the strong hydrogen-bonding interaction between the N atom of pyridine and the L398 backbone hydrogen donor. The NH atom of imidazole showed a common low electron density in the EWG and EDG region, which explains the hydrogen-bonding interaction with the L398 backbone hydrogen acceptor. In other words, the strong electrostatic energy and charge transfer energy between compound **4** and L398 compared to compounds **1**–**3** were evidenced by the electron density distribution of pyridine N atoms in the MEP surface results. In addition, because pyrazole interacts with negatively charged E399, a low electron density was believed to be suitable. However, compound **4** was slightly more positively charged than compounds **1**–**3**, demonstrating repulsion between E399 and compound **4** in the PIE results. From the results of the MEP surface, it was confirmed that the properties of the compound change were owing to atomic substitution and that it affects not only the substituted part but also the region where it interacts with the hinge region. In conclusion, the difference in the biological activity of PAK4 due to the atom-substituted compounds **1**–**4** was explained by the interaction difference between the key residues M395 and K350 and the hinge regions L398 and E399.

## 3. Discussion

Substitution of atoms in the small molecules might, depending on their properties, change their physicochemical properties or affect the bioavailability and efficacy of bioactive molecules [5,6]. Overexpression or activation of the target protein PAK4 is often associated with cancer and oncogenic transformation [9,10,11,12,13,14,15,16,17,18]. In a previous study, we found a small-molecule compound with biological inhibition of PAK4 with a unique scaffold [19]. A structural feature of PAK4 is that the hinge region consists of L398 and E399, and the gatekeeper consists of M395 and K350. The binding mode of compound **1** was revealed through the X-ray crystal structure, and the bromine group of the initial hit compound **1** showed a van der Waals interaction with the aforementioned key gatekeeper residue. Thus, in this study, we used both experimental and in silico methods to investigate the effect of atomic substitution on biological activity to optimize the initial hit compound. To carry out the experimental in vitro bioassay, instead of the bromine group, we purchased compounds substituted with other EWGs such as Cl and F and compounds substituted with CH_3_, the EDG, and performed in vitro assays. The IC_50_ values for each compound from the in vitro assays were above 5150, 8533, and above 30,000 nM for compounds **1** (Br), **2** (Cl), and **3** (F) with EWG, respectively, and above 30,000 nM for compound **4** (CH_3_) with EDG. Therefore, this study aimed to describe the properties of functional groups that affect the biological activity of PAK4 using an in silico method.

We used molecular docking [46] and the FMO method [47] as the main methods for explaining the biological activity and visualized the MEP surface [45]. We also performed the PIE analysis [33] for verification of the FMO method and then analyzed the electron density.

First, molecular docking results revealed that the predicted binding mode was formed, exhibiting hydrogen bonding interactions with the hinge region’s L398 backbone, which was similar for the four compounds containing EWG or EDG. As for the molecular docking score, compound **1**, with an IC_50_ value of 5150 nM, showed the highest score of 59.27, but compound **4**, with an IC_50_ value of above 30,000 nM, showed the second-highest score of 58.14. In addition, the binding energy value of compound **2**, with an IC_50_ value of 8533 nM, was predicted to be the most stable among the four compounds. Therefore, in the case of molecular docking, the results were insufficient to explain the biological activity of PAK4 based on the molecular docking score and/or binding energy.

Second, the FMO method considers electrons and quantitatively calculates the interaction energy between the ligand and fragment by dividing the macromolecule into fragments. In this study, the energy analysis PIE results were divided into M395 and K350 of gatekeeper and L398 and E399 of hinge region residues. For the gatekeeper residues, lower PIE values were observed for compounds **1**–**4**. In the case of compounds **1**–**3** with EWGs, it is explained by the energy difference depending on the size of the substituted atom and the interaction distance between the gatekeeper residues. However, we believe that the interaction energy value between each compound and the gatekeeper residue will not reflect the properties of EWG and EDG. Therefore, we hypothesized that there might be other residues that distinguish the properties of compounds in EWG and EDG. Interestingly, the PIE values of the EWG and EDG showed opposite patterns in the hinge regions L398 and E399. We analyzed the electrostatic energy and charge transfer energy involved in the hydrogen-bonding interactions in the PIE results. The electrostatic and charge transfer energies of L398 and compound **4** interacted strongly with −2.91 and −0.41 kcal/mol values, respectively, compared to compound **1**. Conversely, the electrostatic and charge transfer energies of E399 and compound **4** interacted weakly with 4.41 and 1.33 kcal/mol values, respectively, compared to compound **1**. Based on the PIE results, the difference in biological activity based on the properties of EWG and EDG can be explained by the difference in interaction energy with the hinge region residue. Additionally, we visualized the MEP surface of the compound and observed the electron density in the hinge region and the portion where the interaction was anticipated. The pyridine N atom of the scaffold, which is believed to interact with the hydrogen donor of the L398 backbone, showed a higher electron density in compound **4** than in the other compounds. Moreover, the pyrazole moiety of the compound anticipated to interact with E399 showed a lower electron density in compound **4** than in the other compounds. This result verifies that compound **4** not only has strong interaction energy with L398 in the PIE results but also exhibits repulsion with E399. In this study, we attempted to verify the biological activity of PAK4 using molecular docking and FMO. Verification of the biological activity values of EWG and EDG, which were not explained in molecular docking, was confirmed by the difference in the interaction energy at the hinge region in PIE analysis through FMO calculations. In addition, the PIE results were supported by the electron density at the hydrogen-bonding interaction region through MEP surface calculations. Through this study, we were able to suggest in silico method that can explain even minor changes in the functional group in order to determine the optimal functional group within the specific binding site in the early drug discovery process.

## 4. Materials and Methods

### 4.1. Molecular Docking Study

The X-ray crystal structures of human PAK4 (PDB code 5I0B) [19] were downloaded from the protein data bank (PDB) site (https://www.rcsb.org/structure/5I0B, accessed on: 14 December 2016). The structure was processed using the ‘Prepare Protein of Automatic Preparation’ module of Discovery Studio 2021 (BIOVIA, San Diego, CA, USA). This process included the identification of inserting missing atoms in incomplete residues, modeling missing loop regions, missing residues, the addition of hydrogen atoms, and assignment of bond orders and formal charges. Protonation states were assigned under the assumption that systems had a pH of 7.4. The different atom of compounds was changed in the binding mode of X-ray crystal structure ligand conformation. Energy minimization of compounds was performed using the ‘Full Minimization of Minimization’ module of Discovery Studio 2021. Ligand minimization was performed using the CHARMm force field [48] and smart minimizer algorithm. Partial charges were assigned using the Momany–Rone partial charge method.

After the protein and compound preparation step, molecular docking studies were performed on the processed structures using the LigandFit module [46] of receptor-ligand interactions tools of Discovery Studio 2021 (BIOVIA, San Diego, CA, USA). The binding site was defined based on the co-crystallized ligand. For each ligand, one hundred docked poses were generated for each ligand and scored using scoring functions. Protein-ligand interactions were considered for selecting the binding modes of the ligands.

### 4.2. FMO Calculations

The ligands used in the FMO method were the same as the ligands in the molecular docking study preparation step. The prepared X-ray crystal structure was subjected to a limited minimization procedure using the AMBER99 force field [49] and water model TIP3P [50] in GROMACS version 5.1.4 (Royal Institute of Technology and Uppsala University, Stockholm, Sweden) [51]. Since small errors in atomic positions can be interpreted as large deviations in terms of energy, it is essential not to gap the protein sequence in the structure and proceed with optimization of the structure.

FMO calculations of each compound and PAK4 protein complex were performed with the General Atomic and Molecular Electronic Structure System (GAMESS) software package (Ames Laboratory/lowa State University, Ames, US) [47]. The FMO calculation is a large biological system that is divided into smaller parts called fragments [26,27,52,53]. The analysis proceeded with standard FMO practice of fragmentation of amino acids, including NH and CO linkers [33]. Amino acids are fragmented along the sp3 bond connecting the Cα carbon to the peptide bond carbonyl carbon [54]. Thus, it is possible to explain the interaction energy between each fragmented residue and the compound. The two-body FMO calculation [26,29,55] is composed of four steps: (a) Divide the protein molecule into fragments and assign electrons to the fragments. (b) Monomer fragment self-consistent field (SCF) calculations. (c) Dimer fragment SCF calculations. (d) Total property evaluation such as energy and gradient. We used the Third Order Density Functional Tight Binding (DFTB3) method with Third-Order Parametrization for Organic and Biological Systems (3OB-3-1). This parameter set is part of the 3OB and has been designed for DFTB3 instead of the MIO (parameters for materials and biological systems) parameter set [56,57,58], and we used the 6-31G** basis set. This basis set is commonly used and is often considered the compromise between speed and accuracy [54]. In the FMO calculations, water was used from the Polarizable Continuum solvation Model (PCM), and there was no need to neutralize the charged residues since they can be treated with a PCM level [59]. Residues and water molecules within a radius of 5.0 Å around the ligand atoms were included in the FMO calculations to increase the speed of the calculation [29]. The energy between the two fragments performed the energy decomposition of the PIE based on the contributions of the five energy terms. PIE does not mean the difference between the energy of the protein-ligand complex state and the sum of the energies of the apoprotein and ligand, but rather the “strength” of the interaction between the ligand and the protein residue in the binding state [33]. Energy Equation 1 (Equation (1)) is decomposed into five terms in PIE: electrostatic (Δ*E_ij_^es^*), exchange-repulsion (Δ*E_ij_^ex^*), charge transfer with a higher-order mixed term (Δ*E_ij_^ct+mix^*), dispersion (Δ*E_ij_^di^*), and solvation energy (Δ*G_sol_*) obtained from the PCM. The Δ symbol indicates the difference in total QM energy of fragment pair *ij* and each fragment *i* and *j* calculated in the protein-ligand complex.

(1) $$\Delta E_{ij}^{int}=\Delta E_{ij}^{es}+\Delta E_{ij}^{ex}+\Delta E_{ij}^{ct+mix}+\Delta E_{ij}^{di}+\Delta G_{sol}$$

### 4.3. Quantum Mechanics Chemical Calculations

The theoretical calculations were performed using the Gaussian 09 package [60], and GaussView 5.0 software (Wallingford, CT 06492 USA, https://gaussian.com/, accessed on 23 February 2022) [61] was utilized in the visualization of the Gaussian output files. The compounds in the ground state were optimized using density functional theory (DFT) using the Becke 3 Lee-Yang-Parr (B3LYP) functional at the 6-311G++ (3df) basis set [62,63,64,65]. The electrostatic potential for each compound was mapped onto a total electron density surface. In the MEP surface, the compound’s electrostatic potentials at the surface of the molecule are indicated by different colors. The electrostatic potential expresses an increase in the order of red to blue, with red representing electron-rich regions and blue representing electron-poor regions. The compound electrostatic potential was in the range −1.500 × 10^−2^ to 5.000 × 10^−2^.

### 4.4. In Vitro Assay

Enzymatic assays for PAK4 were performed by Eurofins Scientific Inc. Korea (Brussels, Belgium). KinaseProfiler sevice assay v86 was performed, and the reference inhibitor for PAK4 was staurosporine (9.17 nM IC_50_ value). The assay was performed in duplicate, and the average IC_50_ value was reported.

PAK4 (h) was incubated with 8 mM MOPS pH 7.0, 0.2 mM EDTA, 0.8 mg/mL myelin basic protein, 10 mM magnesium acetate, and [γ-^33^P]-ATP (specific activity and concentration as required). The reaction was initiated by the addition of the Mg/ATP mix. After incubation for 40 min at room temperature, the reaction was stopped by the addition of phosphoric acid to a concentration of 0.5%. An aliquot of the reaction was then spotted onto a filter and washed four times for 4 min in 0.425% phosphoric acid and once in methanol prior to drying and scintillation counting.

### 4.5. Procurement and Synthesis

Compound **1** (6-bromo-2-(3-isopropyl-1-methyl-1H-pyrazol-4-yl)-3H-imidazo[4,5-b]pyridine) was synthesized and characterized as reported in our previous study [19]. Compound **2** (6-chloro-2-(3-isopropyl-1-methyl-1H-pyrazol-4-yl)-3H-imidazo[4,5-b]pyridine), compound **3** (6-fluoro-2-(3-isopropyl-1-methyl-1H-pyrazol-4-yl)-3H-imidazo[4,5-b]pyridine), and compound **4** (2-(3-isopropyl-1-methyl-1H-pyrazol-4-yl)-6-methyl-3H-imidazo[4,5-b]pyridine) were purchased from OTAVA chemicals, Ltd. (Vaughan, Ontario, L4K 0C3, Canada, https://otavachemicals.com/, accessed on 17 April 2007).

## Figures and Tables

**Table 1:** Docking score, binding energy, PIE, and in vitro assay results for compounds **1**–**4** against PAK4.

	1	2	3	4
Docking score	59.27	56.95	54.00	58.14
Binding energy	−23.78	−27.14	−25.16	−22.78
PIE	−22.33	−22.10	−21.39	−16.30
IC_50_ (nM)	5150	8533	>30,000	>30,000

**Table 2:** Pair interaction energies (E^int^) calculated at the FMO-DFTB3/3OB-3-1 level. The pair interaction energies (E^int^) of the hinge regions, L398 and E399, are shown in bold.

	Residue	I372	G328	V335	I337	A348	K350	V379	M395	E396	F397	L398	E399	G400	G401	A402	L403	D405	L447	S457	D458
Compound																					
**1**		−0.45	−1.37	−0.75	1.35	−2.99	−9.84	−1.66	−2.33	4.84	−0.25	**−2.88**	**−3.48**	−3.88	5.08	−2.55	−0.71	−1.70	−0.99	−1.67	3.88
**2**		−0.45	−1.35	−0.74	1.29	−3.35	−9.22	−1.76	−2.06	4.68	−0.24	**−3.00**	**−3.15**	−3.83	4.92	−2.46	−0.68	−1.69	−1.02	−1.69	3.70
**3**		−0.32	−1.37	−0.62	1.32	−2.87	−8.98	−1.68	−1.54	4.38	−0.16	**−2.72**	**−3.85**	−3.92	5.14	−2.52	−0.75	−1.99	−0.83	−1.40	3.29
**4**		−1.20	−1.21	−1.28	0.89	−3.86	−6.28	−1.78	−1.20	5.09	−0.62	**−6.57**	**2.74**	−3.29	3.65	−2.64	−0.47	−0.82	−1.94	−2.21	4.19

**Table 3:** Electrostatic energies (E^es^) calculated at the FMO-DFTB3/3OB-3-1 level. The Electrostatic energies (E^es^) of the hinge regions, L398 and E399, are shown in bold.

	Residue	I372	G328	V335	I337	A348	K350	V379	M395	E396	F397	L398	E399	G400	G401	A402	L403	D405	L447	S457	D458
Compound																					
**1**		−0.24	−2.30	−0.97	1.64	−3.47	−12.30	−1.58	−1.24	5.18	−0.01	**−4.37**	**−8.26**	−5.37	3.87	−2.54	−1.67	−5.82	−1.37	−1.62	6.89
**2**		−0.24	−2.26	−0.97	1.58	−3.43	−11.53	−1.56	−1.23	5.09	−0.01	**−4.44**	**−7.92**	−5.29	3.71	−2.45	−1.62	−5.69	−1.39	−1.65	6.73
**3**		−0.17	−2.33	−0.90	1.69	−3.48	−12.83	−1.62	−1.30	5.27	0.22	**−4.24**	**−8.30**	−5.40	3.90	−2.49	−1.71	−5.90	−1.28	−1.61	6.95
**4**		−0.86	−1.99	−1.50	1.11	−4.02	−6.48	−1.58	−1.06	4.75	−0.60	**−7.28**	**−3.85**	−4.86	2.71	−2.71	−1.40	−4.77	−2.27	−2.35	5.82

**Table 4:** Charge transfer energies (E^ct^) calculated at the FMO-DFTB3/3OB-3-1 level. The Charge transfer energies (E^ct^) of the hinge regions, L398 and E399, are shown in bold.

	Residue	I372	G328	V335	I337	A348	K350	V379	M395	E396	F397	L398	E399	G400	G401	A402	L403	D405	L447	S457	D458
Compound																					
**1**		0.00	−0.01	0.00	0.00	0.03	0.06	−0.01	−0.05	0.00	−0.06	**0.02**	**−2.28**	−0.16	0.05	−0.03	0.00	0.00	0.01	−0.03	0.00
**2**		0.00	−0.01	0.00	0.00	0.09	0.13	−0.03	−0.03	0.00	−0.06	**−0.01**	**−2.18**	−0.16	0.05	−0.03	0.00	0.00	0.01	−0.03	0.00
**3**		0.00	−0.01	0.00	0.00	0.00	0.00	0.00	0.00	0.00	−0.06	**−0.01**	**−2.15**	−0.16	0.05	−0.03	0.00	0.00	0.01	0.00	0.00
**4**		−0.01	−0.01	−0.01	0.00	0.00	0.00	0.00	0.00	0.00	−0.02	**−0.39**	**−0.95**	−0.10	−0.05	−0.02	0.00	0.00	−0.01	0.01	0.00
